# Efficacy of Intrauterine Device in the Treatment of Intrauterine Adhesions

**DOI:** 10.1155/2014/589296

**Published:** 2014-09-01

**Authors:** Umme Salma, Min Xue, Ali Sheikh Md Sayed, Dabao Xu

**Affiliations:** ^1^Department of Gynecology, Third Xiangya Hospital, Central South University, 138 Tongzipo Road, Changsha, Hunan 410013, China; ^2^Department of Cardiology, Xiangya Hospital, Central South University, Changsha, China

## Abstract

The primary purpose of this paper is to assess the efficacy of the use of the intrauterine device (IUD) as an adjunctive treatment modality, for intrauterine adhesions (IUAs). All eligible literatures were identified by electronic databases including PubMed, Scopus, and Web of Science. Additional relevant articles were identified from citations in these publications. There were 28 studies included for a systematic review. Of these, 5 studies were eligible for meta-analysis and 23 for qualitative assessment only. Twenty-eight studies related to the use of IUDs as ancillary treatment following adhesiolysis were identified. Of these studies, 25 studies at least one of the following methods were carried out as ancillary treatment: Foley catheter, hyaluronic acid gel, hormonal therapy, or amnion graft in addition to the IUD. There was one study that used IUD therapy as a single ancillary treatment. In 2 studies, no adjunctive therapy was used after adhesiolysis. There was a wide range of reported menstrual and fertility outcomes which were associated with the use of IUD combined with other ancillary treatments. At present, the IUD is beneficial in patients with IUA, regardless of stage of adhesions. However, IUD needs to be combined with other ancillary treatments to obtain maximal outcomes, in particular in patients with moderate to severe IUA.

## 1. Introduction

Intrauterine adhesions (IUAs) or Asherman's syndrome has been reported and studied for more than a century. This disease occurs mainly as a result of the trauma of dilatation and curettage, postabortal infection, hypoestrogenism, genital tuberculosis, and previous uterine surgery, producing partial or complete obliteration in the uterine cavity and/or the cervical canal, resulting in conditions such as amenorrhea, hypomenorrhea, infertility, or recurrent pregnancy loss [1–7]. Despite the wide use of diagnostic and operative hysteroscopy, the management of Asherman's syndrome is still challenging [7]. Many studies have reported on the reproductive outcome after treatment of IUA [8–16]. Hysteroscopy represents the gold standard method for the definitive diagnosis and treatment of the IUA. The aim of treatment of IUA is to restore a normal uterine cavity, resume normal menstruation, and improve pregnancy outcomes [17]. The ideal treatment of IUA consists not only of physically removing the adhesion but also of preventing the formation of new ones by the use of other adjunctive measures. Currently most surgeons have recommended that intrauterine readhesion is prevented by using an IUD [18]. The placement of an IUD in the uterine cavity has been the standard method of maintaining the uterine cavity and frequently is used for the prevention of subsequent adhesion formation after adhesiolysis [4, 19]. It was speculated that an IUD could help the physiological endometrial regeneration by separating the anterior and posterior uterine walls, although many authors have reported good results [20, 21]. Many investigators support the use of IUDs (especially the Lippes loop) for prevention of recurrent IUAs [2, 17, 22–24]. Other studies reported that copper-bearing and Progestasert (Alza Corporation, Palo Alto, CA) IUDs may have a rather small surface area and may not be able to prevent adhesion reformation. Besides, copper-bearing IUDs may induce an excessive inflammatory reaction. It is thought that the placement of an IUD would help to keep opposing surfaces of the uterine cavity separation and subsequent removal of the IUD may also help to remove some adhesions which may have reformed [25]. Some investigators reported that the IUD may provoke local inflammation and increase the likelihood of reformation of adhesions [25, 26]. This treatment remains empirically based. In previous studies of IUA, the various protocols of IUD therapy have been used in terms of the types of IUD, duration of course, and combination of hormones and other ancillary methods. Our primary objective of this study is to highlight the efficiency of intrauterine device (IUD) as an adjunctive treatment modality, for management of IUAs.

## 2. Methods

This systematic review and meta-analysis was conducted in accordance with PRISMA (preferred reporting items for systematic reviews and meta-analyses) guidelines.

### 2.1. Search Strategy

All eligible studies were identified on computerized databases (PubMed, Scopus, and Web of Science), using the keywords “Asherman syndrome,” “Asherman's syndrome,” “Fritsch syndrome,” “gynatresia,” “intrauterine adhesions,” “intrauterine synechiae,” “synechia uteri,” and “uterine synechiae.” The search included studies from the earliest publication date to February 2014 in English publications but some IUD use in Chinese patent that translated to English. Additional relevant articles were identified from citations within these publications.

### 2.2. Study Characteristics

Because of the lack of randomized control trials (RCT), observational studies (prospective/retrospective cohort and case-control studies) were included for review. Reviews and case reports were excluded from this systematic review. Studies were selected by electronic databases including PubMed, Scopus, and Web of Science. First, eligibility was assessed based on the title and abstract. Full manuscripts were obtained for all studies that were selected. In the second step, examination of the full manuscript was carried out to study the eligibility of the study. Most of the studies used multiple ancillary treatment methods to prevent readhesions in the treatment of IUAs. There was no single study that was solely focused on comparing the efficacy of IUD as an adjunctive therapy in patients with IUAs following the adhesiolysis procedure. Therefore, we evaluated and examined the outcomes of all included studies that used the following various techniques of the adhesiolysis procedure such as Foley catheter, hyaluronic acid gel, hormonal therapy, or amnion graft in addition to the IUD.

### 2.3. Outcomes

The primary outcomes measure of the IUDs as ancillary treatment following adhesiolysis was identified with the management of IUAs. Secondary meta-analyses were performed to estimate the association between outcomes of IUA with relation of classification of IUA, type of IUD, and duration course of IUD. Subsequently in the second analysis for menstruation, pregnancy, and live birth rates.

### 2.4. Data Extraction and Statistical Analysis

From each study, the following data was extracted: first author, year of publication, type of study, classification of IUA, the number of participants, mean age, stage of adhesion, surgical techniques of adhesiolysis, type of IUD, duration course of IUD, ancillary treatment used (hormone therapy, Foley catheter, hyaluronic acid gel, and amnion graft), and complications. The primary outcomes of interest included clinical outcomes (normal or improvement in menstrual flow, pregnancy, and live birth rates). Studies were eligible for meta-analysis if the methods of follow-up were adequate for the outcome and necessary statistics could be retrieved.

Statistical analyses were performed by using the Review Manager (RevMan) version 5.0 software (The Cochrane Collaboration, Copenhagen, Denmark) and SAS 9.3 (SAS Institute, Cary, NC, USA). The Mantel-Haenszel method was conducted for pooling of dichotomous data and presented as odds ratio (OR) with 95% confidence interval (CI). The presence of statistical heterogeneity was calculated using the *I* ^2^ statistics. Heterogeneity was measured substantially when *I* ^2^ was ≥50%. In order to compare the overall outcomes of IUAs in women following Lippes loop IUD with 3-month follow-up in the treatment of IUA, pooled OR and 95% confidence intervals (CI) were calculated. Depending on the presence of statistical heterogeneity, the data of studies were pooled on the basis of a fixed effects model or a random effects model. *P* < 0.05 was considered to be statistically significant.

## 3. Results

### 3.1. Identification and Selection of Literature

**Figure 1:**
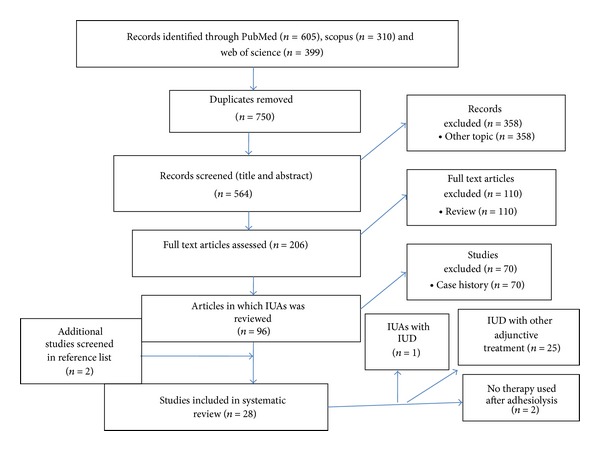
Flow chart showing search results.

Searches identified 1314 publications. The search strategy yielded 605 from PubMed, 310 from Scopus, and 399 from Web of Science citations including 750 duplicates. A flow chart showing search results appeared in Figure 1. There were 564 potentially relevant articles identified from title and abstract. Of the 564 articles, 358 were not relevant, 110 were reviews, and 70 were case reports. After applying exclusions, 26 studies [7–9, 11–13, 16, 18, 19, 27–42] were eligible for this systematic review. Of these studies, one study [43] included IUD therapy as a single ancillary treatment and 25 studies used IUD with Foley catheter, hyaluronic acid gel, hormonal therapy, or amnion graft. Two additional studies [44, 45] were identified from citations in these publications and were included for review. Among them 5 studies [12, 19, 30, 32, 33] were eligible for meta-analysis due to the same applied type of IUD (Lippes loop) and 3-months-follow-up. 23 studies remain for qualitative assessment only due to variable type of IUD and duration of follow-up.

### 3.2. Description of Included Studies

Characteristics of the included studies are given (Table 1). Various classification systems of IUA staging used in the studies identified made unification of results from these studies more challenging such as March et al. [46], European society classification [47], the American Fertility Society classification [48], Valle and Sciarra [8], Donnez and Nisolle classification [49], and, very recently, Aboul Nasr et al. [50]. No one of these classification systems has been validated by clinical studies, and no one has used them uniformly when reporting outcome after treatment of intrauterine adhesions. Thus, comparisons among the different reports that include outcomes are difficult. Of the 28 studies, 5 [12, 19, 30, 32, 33] studies were included in the meta-analysis with characteristics of the same used type of IUD (Lippes loop) with 3-month follow-up in the management of the IUA. Characteristics of the included studies are given (Table 1). In our systematic review the classification systems used included (Table 2) those from the American Fertility Society [9, 16, 27, 28, 34–36, 41, 44, 45], European Society of Gynaecological Endoscopy [7, 29, 39], American Society for Reproductive Medicine [11], European Society of Hysteroscopy [29, 38], European Society of Human Reproduction and Embryology [45], modified Sugimoto criteria [13], and the March classification system [18]. Three studies [7, 29, 45] reported the stages of adhesion using 2 classification systems; however, several studies [8, 12, 19, 30–33, 37, 42, 43] did not provide any information of the classification system used. Twelve studies identified outcomes in mild to severe IUA [7, 8, 11, 16, 27, 29, 33, 36–40], 4 studies in severe IUA [18, 28, 34, 35], 1 study in mild to moderate IUA [43], and 1 study in moderate to severe IUA [41]. A total of 1806 patients were studied. The number of participants in each study varied from 7 to 365 (mean, 56.96). Participants were aged 21 to 48 years old. In twenty-six studies the IUD was used as ancillary treatment after adhesiolysis. Twenty-five studies included at least one of the following methods: Foley catheter, hyaluronic acid gel, hormone therapy, or amnion graft as ancillary treatment [7–9, 11–13, 16, 18, 19, 27–42]. One study [43] included IUD as a single ancillary treatment. In 2 studies [44, 45], no therapy was administered after adhesiolysis. Surgical instruments and techniques used for adhesiolysis included uterine sound [19, 39], with uterine dilators [7, 29, 32], mechanical D&C [44], hysteroscopic scissors [9, 13, 16, 18, 34, 35, 38–40], hysteroscopic, monopolar, or bipolar knife/needle [29, 34, 39, 45], and bipolar electrosurgery system [7, 16, 39, 45]. Twenty-five studies [7–9, 11–13, 16, 18, 19, 27–42] used IUD in combination with at least one other ancillary treatment. These studies reported menstrual improvement rates between 60% and 100%. One study that used IUD alone as ancillary treatment resulted in restoration of menstrual flow rate that was 90%. Two studies [43, 45] did not use any therapy after adhesiolysis, and one of them [46] reported a menstrual improvement rate of 4.3%, after 2 surgical procedures. Insofar as fertility outcomes, a wide range of pregnancy and live birth rates were reported. A study that used IUD alone as an ancillary treatment reported pregnancy rate of 90% and live birth rate of 85% [43]. Studies that used a combination of IUD and other ancillary treatments reported pregnancy rates between 8% [32] and 100% [31] and live birth rates between 5.2% [32] and 100% [31]. Studies that did not use any therapy after adhesiolysis [44, 45] reported pregnancy rates of 40.9% and 42.5%, respectively, and live birth rates of 27.27%. Despite good results, this method has been associated with several complications such as uterine perforations [7, 8, 13, 39], genital sepsis [8, 16, 19], and urinary tract infections [13, 19]. Obstetric complications included placenta accreta or percreta [7, 16, 29] and postpartum hemorrhage [7].

### 3.3. Outcomes

**Figure 2:**
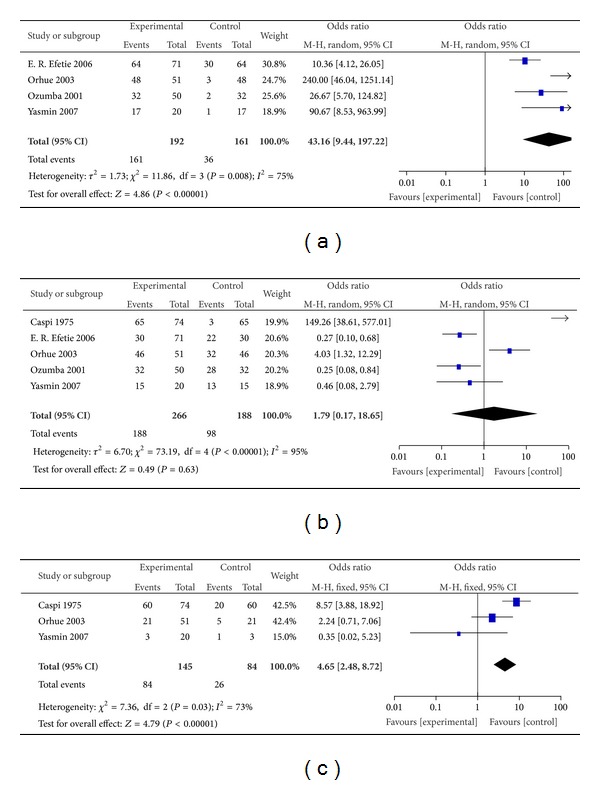
Summary of meta-analysis presenting odds ratio (OR) with 95% confidence interval (CI) for menstruation rates number (a), fertility rates number (b), and live birth rates number (c) of postoperative use of Lippes loop IUD with 3-month follow-up for the management of IUAs. IUAs: intrauterine adhesions and IUD: intrauterine device.

The meta-analysis results are summarized in Figure 2. We calculated the total data of five studies in IUAs patients which compared them after use of IUD. In four studies, there were 161 cases of IUAs among 192 women with abnormal menstruation compared after use of IUD with 36 cases of IUA among 161 women with abnormal menstruation (Figure 2(a)), OR 43.16 (95% CI: 9.44–197.22), *I* ^2^ = 75%, and *P* ≤ 0.00001. The menstruation rates of postoperative use of IUD were statistically significant.

In five studies, there were 188 cases of IUAs among 266 women with secondary infertility compared after use of IUD with 98 cases of IUA among 188 women with secondary infertility (Figure 2(b)), OR 1.79 (95% CI: 0.17–18.65), *I* ^2^ = 95%, and *P* = 0.63. The fertility rates of postoperative use of the IUD were not statistically significant.

In three studies, there were 84 cases of IUAs among 145 women with abortion compared after use of IUD with 26 cases of IUA among 84 women with abortion (Figure 2(c)), OR 4.65 (95% CI: 2.48–8.72), *I* ^2^ = 73%, and *P* ≤ 0.00001. The live birth rates of postoperative use of IUD were statistically significant.

## 4. Discussion

### 4.1. Efficacy of IUD in Patients with IUA

Postoperative use of the intrauterine device (IUD) as an adjunctive treatment modality, for intrauterine adhesions (IUAs). IUD needs to be combined with other ancillary treatments to obtain maximal clinical outcome (improvement in menstrual flow) and fertility (pregnancy and live birth rates), in particular in patients with moderate to severe IUA. Because of the high rate of reformation of intrauterine adhesions (3.1% to 23.5%), especially severe adhesions (20% to 62.5%), preventing of reformation of adhesions after surgery is essential to successful treatment [7, 29]. Various methods have been used to achieve this aim.

Hysteroscopic adhesiolysis [51] causes improvement of endometrial thickness and reepithelization that reported improvement of amenorrhea or hypomenorrhea. Hysteroscopic technique used electrode with the goal of widening the endometrial cavity and promoting endometrial regrowth [52]. Insertion of an IUD provides a physical barrier between the uterine walls and IUD keeps the raw, dissected surfaces separated during the initial healing phase and may reduce the chances that they will readhere to one another [2, 28, 46, 53]. The IUD has been advocated by many studies as an effective, widely used method to prevent adhesion reformation [2, 21, 36–38]. Previously, the Lippes loop IUD was favored for prevention of adhesions due to its large surface area and otherwise inert qualities; however, this device is no longer available in the United States. Currently available devices are T-shaped and include those impregnated with progestin, which suppress endometrial proliferation. Both of these are suboptimal in preventing intrauterine adhesions. The investigators attributed this effect to the inflammatory reaction stimulated by copper IUDs in the endometrium as a consequence of releasing of various types of prostaglandins and enzymes. In a literature review, March [53] discussed the use of IUD and concluded that T-shaped IUD may have too small surface area to prevent adhesion reformation and that IUD containing copper may induce an excessive inflammatory reaction. Therefore, their use is not advised in patients who have had intrauterine adhesions. IUD has been reported as an adjunctive treatment in many studies [7–9, 11–13, 16, 18, 19, 27–42].

**Figure 3:**
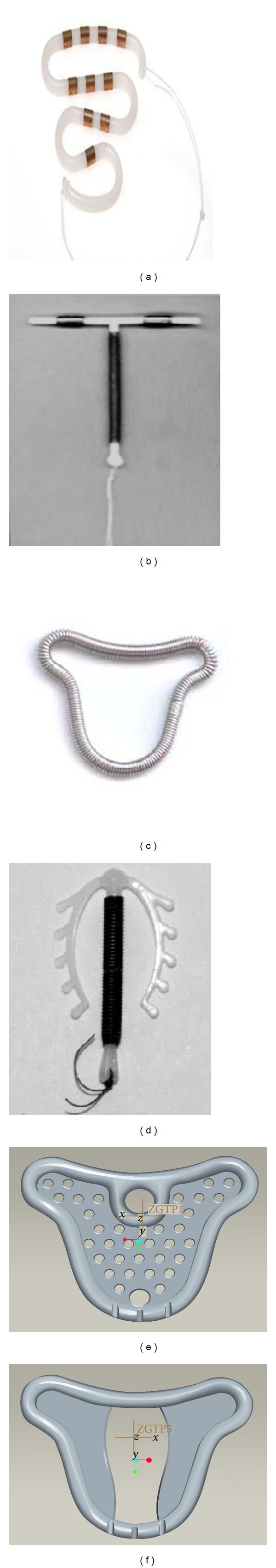
Types of IUD. (a) Lippes loop (patent number US3802425 A). Many investigators support the use of a Lippes loop of IUD. (b) T-shaped (patent number US4026281 A). IUDs are thought to have too small surface area to be truly effective in providing a physical barrier. (c) Uterine-shaped (patent number CN201220343083) IUD. The uterine-shaped IUD was originally manufactured in Chongqing, Sichuan. It is designed in the shape of the uterine cavity, consisting of a stainless steel coiled wire framework with copper added inside the coil wire, and releases anti-inflammatory agent. The uterine-shaped IUD is the most commonly used IUD in China. (d) Multiload Cu 375 (patent number EP2198815 A1). This IUD consists of a copper-bearing plastic shaft and two small flexible curved side arms. Some authors suggested that the copper-containing IUDs provoke an inflammatory reaction. (e) Recently, a new type of uterine-shaped IUDs was researched and manufactured in China with China patent number Zl 2008 2 0052366.3 and (f) another new product with a China patent number Zl 2012 20070407.8; this type of devices is only used for IUA.

Uterine-shaped IUD [34] consists of a stainless steel coiled wire with copper added inside the coil wire and releases anti-inflammatory agent. This type of IUD (uterine-shaped) is frequently practiced in China and getting good outcomes comparable to USA or Europe that usually practice copper type of IUD (CuT). However the specific type to be used for this purpose remains a controversial issue. American Association of Gynecologic Laparoscopists (AAGL) in its practice guideline on IUA has also suggested the use of postoperative IUD to reduce recurrence of IUAs [54]. Schenker and Margalioth [2] used a brief combination of the placement of an IUD therapy after curettage. The placement of an IUD in the uterine cavity for 3 months has considered the standard method of maintaining the uterine cavity after uterine forced intervention [7–9, 12, 13, 19, 30, 32, 33, 38–42, 42, 43]. Different studies preferred different duration courses of IUD such as 1, 2, and 3 months [7–9, 11–13, 16, 18, 19, 27–42]. However, the specific type and duration course to be used for this purpose remain a controversial issue. Our data are conflicting, and there is also uncertainty about the type and duration course of IUD to be used. Over the last two decades of IUD use, a number of studies have been performed with various types. The IUD has been recommended, including the types “Massouras duck foot” [23], Y-shaped [2, 55], Lippes loop [12, 19, 28, 30, 33, 38], CuT, multiload Cu 375 [7, 13, 16, 18, 29, 35, 38, 39, 42], Cu coil [27], and uterine-shaped IUD [34], placed after hysteroscopic adhesiolysis (Figure 3). Recently, a new type of uterine-shaped IUDs was researched and manufactured in China with China patent numbers Zl 2008 2 0052366.3 and Zl 2012 20070407.8 (Figures 3(e) and 3(f)). These two devices were only used for treatment of IUA and they are still under experiment. A summary of previous published studies that used different techniques of IUD in patients with IUA (Table 3). Up to now, there have been no randomized, controlled trials to confirm the usefulness of the exert type and duration course of the IUD for preventing adhesion reformation after hysteroscopic lysis of intrauterine adhesions.

### 4.2. Restoration of Menstrual Flow

Improvement of menstrual blood flow is the end result in most cases of adhesiolysis varying from 88.2% to 100%. The rate of restoration of menstrual flow was 4.3%, after 2 surgical procedures, in studies that did not use IUD, 90% in a study that used IUD alone, and 60% to 100% in studies that used IUD in combination with other ancillary treatments. Normal menstruations were restored in over 90% of the patients following lysis of the IUA (Table 1). However the copper IUD placed after hysteroscopic lysis of adhesion was found to restore normal menses in 40 of 48 women [40]. Valle and Sciarra reported rate resuming normal menstruation of 88.2% [8]. Orhue et al. found that IUD therapy was effective, with 73% of women experiencing a return of menstruation [19]. From nineteen available studies, we can conclude that, of 1000 women who underwent surgical treatment of intrauterine adhesion, 797 of 1000 (79.7%) regained normal menstruation. In four [7, 27, 29, 36], studies reported improved menstrual flow in 134 of 192 (69.7%). However two studies [44, 45] did not use any treatment or adhesion barrier to prevent recurrent adhesions. Fumino et al. [44] reported satisfactory results, with 35 of 47 patients free of adhesions at second-look hysteroscopy and 7 patients free of adhesions after a third hysteroscopy. Menstrual blood flow was not restored in only 5 patients who had dense adhesions before the procedure [44]. This promising result was contradicted by Fernandez et al. [45], who found that, with the surgical procedure alone, only 1 of 24 women (4.3%) had normal menstrual cycles after 2 surgical procedures. Furthermore, in the remaining 23 patients, more than 2 surgical procedures were necessary: 3 procedures in 12 patients, 4 procedures in 9, and 5 procedures in 2 [45]. These 2 studies emphasized the importance of surgical adhesiolysis and suggest that a satisfactory result could be obtained only by performing repeated surgical procedures. Although it is important, it is known that the adhesiolysis procedure itself is also considered an intervention that causes additional or new trauma to the endometrium and may worsen the regeneration process of the endometrium. All studies that used IUD therapy in combination with other ancillary treatments reported normal and improved menstrual rates that were 70.6% to 100% and 28.5% to 100%, respectively. Thus, IUD therapy may produce a beneficial effect in patients with IUA.

### 4.3. Pregnancy and Live Birth Rates

Insofar as fertility outcomes, a wide range of pregnancy and live birth rates were reported. With respect to fertility, March and Israel's [31] review of numerous studies placed the postoperative pregnancy rates between 60% and 75%, although Valle and Sciarra report a rate of 93% in those with minimal disease [8]. Pregnancy rates are encouraging, but the true measure of reproductive success is viable births. Valle and Sciarra report term pregnancy rates of 55.6% in patients initially presenting with severe adhesions and 87.5% in patients who initially had mild disease [8]. Schenker and Margalioth's [2] findings correlate well with Valle and Sciarra's findings. They note a 95% pregnancy rate with a 15% abortion rate in patients who had mild adhesions and a 60% pregnancy rate with a 50% abortion rate in patients initially presenting with severe disease [2]. Reproductive outcomes correlate well with the type of adhesions and the extent of uterine cavity occlusion. Yu et al. [7] and Roy et al. [29] showed that the conception rates in women with IUA, depending on the stage of adhesions, were 64.7% and 58%, respectively, in patients with mild adhesions, 53.6% and 30% in those with moderate adhesions, and 32.5% and 33.3% in those with severe adhesions. Roy et al. [29] reported a decreasing live birth rate from mild to severe IUA (mild 94.4%, moderate 83.3%, and severe 66.6%). Orhue et al. [19] found that IUD with other ancillary methods was effective, with 73% of women experiencing a return of menstruation and 31% conceived, and the term birth rate was 16%. Studies that used a combination of IUD and other ancillary treatments reported pregnancy rates of 8% to 100% and live birth rates of 5.2% to 100%. Pregnancy rates of 40.9% to 42.5% and live birth rate of 27.27% were found in the studies in which IUD therapy was not used [44, 45]. All of the studies that used IUD with other methods for ancillary treatment reported relatively good pregnancy and live birth rates. However the specific type and duration course of the IUD that can improve fertility outcomes need to be studied further. Notably, pregnancy and live birth rates were greatly influenced by the stage of adhesions. On the other hand, the wide range of clinical diversity in the techniques used for adhesiolysis and methods used for ancillary treatment, IUD therapy combined with other ancillary treatments, could not be compared. Furthermore, the specific type and duration course of IUD that will exert the most beneficial effect remain unknown because there were no studies that compared them with stages of adhesions. The management of moderate to severe adhesions is challenging, and the prognosis of severe disease remains poor.

### 4.4. Comparison with Previous Research

Intrauterine adhesions occur after trauma of the basalis layer of the endometrium generally after endometrial curettage. It was first described by Heinrich Fritsch in 1894 and subsequently studied by Israeli gynecologist Asherman [1, 46]. Hysteroscopy is the current method of the choice of diagnosing, treating, and following patients with Asherman's syndrome [9]. Hysteroscopic adhesiolysis are performed directly or under fluoroscopic guidance [56], laparoscopic guidance [8, 29], or ultrasonographic guidance [35]. Monopolar [8, 14, 57–59], bipolar [16, 45], and electrosurgical instruments and the Nd-YAG laser [8, 58, 59] have been described as techniques used to lyse adhesions under direct vision, with the advantages of precise cutting and good hemostasis. Recently, a vaginoscopic approach to hysteroscopy was introduced to reduce discomfort and pain and also to avoid trauma during removal of IUD [60–62].

Intrauterine adhesion shows endometrial fibrosis in which the stroma is largely replaced with fibrous tissue and the glands are replaced by inactive cubocolumnar endometrial epithelium. The functional and basal layers are indistinguishable, with the functional layer replaced by an epithelial monolayer unresponsive to hormonal stimulation and fibrotic synechiae forming across the cavity [63].

The novel IUD causes local release of cytokines (such as growth factors), cytokines best known for their chemoattractive properties, attract leucocytes into tissues and are present in many leucocytes and endometrial epithelial, stromal, and vascular cells. Evidence now supports a broad range of functions for chemokines would play a positive role in the growth of exterior endometrial stem cells and final regeneration of functional endometrium [22, 64].

Postoperative adhesion formation occurs in almost 50% of the most severe cases and in 21.6% of the moderate ones [8]. Mild synechiae appeared to be the exception in the fact that they tend not to recur. Several approaches have been described for the prevention of adhesion formation [14, 56, 65] with no clear consensus on the postoperative regimen of choice. Polishuk et al. [66] reported that, by following adhesiolysis with IUD placement, the rate of adhesion reformation was only 10%. In contrast, in a prior series of patients treated without an IUD, the recurrence rate was above 50%.

After hysteroscopic adhesiolysis the healed process occurs, with 96% of the women completing their wound healing within 2 months and subsequently endometrium reepithelization, or after hormone treatment to stimulate the endometrium and promote reepithelization [40, 56, 67, 68]. Postoperative readhesion formation was important factor that disturbed the endometrial wound healing. It has been reported to occur after hysteroscopic adhesiolysis because wounds were prone to adhere to each other during the endometrial rebuilding; these have effects later on reproductive outcome [7, 59]. Placement of IUD acts as a temporary mechanical barrier that keeps tissue surfaces separated during the early days of wound healing, when adhesions form and IUD gets facility for the healing process [62, 69]. Duration of endometrial healing depends on the severity of IUA in women following adhesiolysis [70]. IUD has been reported as an adjunctive treatment in many studies [7–9, 11–13, 16, 18, 19, 27–43]. Many investigators support the use of IUDs (especially the Lippes loop) for prevention of recurrent IUAs [2, 17, 22–24]. Other studies reported that copper-bearing and Progestasert (Alza Corporation, Palo Alto, CA) IUDs may have a rather small surface area and may not be able to prevent adhesion reformation. Besides, copper-bearing IUDs may induce an excessive inflammatory reaction. The copper devices increase menstrual blood loss and PGs (prostaglandins) that might be implicated in the pathogenic mechanism through an effect on vascular tone and platelet aggregation [71]. These considerations, together with the increase in menstrual flow observed in women with normal cycles, and after investigating findings that the copper IUD can be used effectively to restore menstrual flow in the management of functional secondary amenorrhea [71].

Hormonal coil which releases a progestin into the endometrium prevents the desired proliferation produced by the postoperative oestrogen therapy. Therefore, its use is not advised. Uterine-shaped IUD [34], concurrent with the reduction in the use of the stainless steel type of IUD and consisting of a stainless steel coiled wire framework with copper added inside the coil wire; this type of device is also releasing anti-inflammatory agent. The placement of an IUD in the uterine cavity for 3 months has considered the standard method of maintaining the uterine cavity after uterine forced intervention [7–9, 12, 13, 19, 30, 32, 33, 38, 40, 42, 43]. Different studies preferred different duration courses of IUD such as 1, 2, and 3 months [7–9, 11–13, 16, 18, 19, 27–43]. However, the specific type and duration course to be used for this purpose remain a controversial issue. The results of treatment after surgical treatment of IUA are promising overall, with the caveat that the severity of the adhesions significantly influences the outcome of treatment, both in terms of recurrence and in terms of symptom resolution. Acceptable anatomical results are usually obtained after postoperative insertion of IUD [66]. Postoperative management of IUA with IUD and the resolution rate for menstrual disorders are between 75 and 100% [2, 9, 14, 16, 37]. Better outcomes are seen in the setting of amenorrhea compared with hypomenorrhea [9], as improvement of the latter fertility outcome. According to Valle and Sciarra comprehensive review of 187 women with Asherman's syndrome [8], the overall pregnancy rate after adhesiolysis was 76.4%, the live birth rate was 79.2%, and resuming normal menstruation rate was 88.2% [8]. Orhue et al. [19] found that IUD with other ancillary methods was effective, with 73% of women experiencing a return of menstruation and 31% conceived, and the term birth rate was 16%. Studies that used a combination of IUD and other ancillary treatments reported pregnancy rates of 8% to 100% and live birth rates of 5.2% to 100%. As mentioned above, the studies on Asherman's syndrome are difficult to compare due to differences in patient selection, classification, and treatment. With respect to pregnancy and live birth rates, success appears to be related to the severity of the adhesions. In a large series, pregnancy rates of 93, 78, and 57% were achieved after treatment of mild, moderate, and severe adhesions, respectively, and these pregnancies resulted in live birth rates of 81, 66, and 32%, respectively [8]. Up to now, there have been no randomized, controlled trials to confirm the usefulness of the exert type and duration course of the IUD for preventing adhesion reformation after hysteroscopic lysis of intrauterine adhesions.

### 4.5. Recommendations for Future Direction

The present systematic review and meta-analysis provides details and literature evidence of IUDs for the management of IUAs patients. However, some limitations of the present paper need to be acknowledged. Firstly, there is lack of appropriate clinical data regarding IUDs for the therapeutic approach of IUA patients. Secondly, most of the findings were based on a single-center study using small samples with different types of IUDs, which lead to many divergences between different reports. Therefore, it is necessary to conduct independent and large cohort studies to identify those IUDs with real value for the prevention of IUA after hysteroscopic adhesiolysis. Future research should focus on cellular and molecular aspects of endometrial tissue about the safety and efficacy of the new invented specific IUDs. These studies should provide an evidence based answer to the ideal IUD, the duration of course therapy, and the stage of adhesions in which IUD therapy will be most beneficial.

## 5. Conclusion

Hysteroscopic management of IUAs is a safe and effective method that ensures lysis of all adhesions. The IUD could be applied after hysteroscopic adhesiolysis to avoid regeneration of IUAs. It seems that IUD needs to be combined with other ancillary treatments such as hormone therapy, Foley catheter, hyaluronic acid gel, or amnion graft to obtain maximal outcomes, particularly in patients with moderate to severe IUA. Placement of an IUD to maintain the uterine cavity is safe and effective in ensuring the return of normal menstruation and later pregnancies with minimal complications. Several studies reported different postoperative outcome after using the IUD; however, no comparative studies have confirmed the ideal IUD, duration course of IUD therapy, and the combination of IUD. Therefore, well-designed prospective, randomized, multicentered clinical trial will be needed to evaluate the potential therapeutic outcome of IUD for the management of intrauterine adhesions.

## Figures and Tables

**Table 1:** Characteristics of included studies.

Source, year	Study type	Number of patients (mean age)	Classification	Adhesion stage	Surgical technique	IUD	HT	FC	Normal/improved menses (%)	Conception rate (%)	Live birth rate (%)
Caspi and Perpinial, 1975 [30]	NR	80 (74 followed up) (NR)	NR	NR	Vaginal approach (long curved scissors)	Yes	Yes	No	NR	62/74 (83.7)	40/62 (64.5)

March and Israel, 1976 [31]	NR	10 (27.1)	NR	NR	Hysteroscopic miniature scissors	Yes	Yes	Yes	10/10 (100) normal	1/1 (100)	1/1 (100)

March and Israel, 1981 [37]	NR	38 (NR)	NR	Mild (*n* = 7) Moderate (*n* = 20) Severe (*n* = 11)	Hysteroscopy with miniature scissors	Yes (35)	Yes	Yes	NR	(87.2)	(87.2)

Ismajovich et al., 1985 [43]	NR	51 (NR)	NR	Mild (*n* = NR) Moderate (*n* = NR)	Hysteroscopic scissors, uterine dilator	Yes	No	No	46/51 (90) normal	46/51 (90)	40/46 (85)

Fedele et al., 1986 [40]	Retrospective	31 (NR)	NR	Mild (*n* = 13) Moderate (*n* = 10) Severe (*n* = 8)	Hysteroscopic scissors	Yes	Yes	No	21/31 (67.7) normal	13/27 (40.7)	13/27 (40.7)

Valle and Sciarra, 1988 [8]	Retrospective	187 (NR)	AFS	Mild (*n* = 43) Moderate (*n* = 97) Severe (*n* = 47)	Hysteroscopy and sharp dissection with hysteroscopic scissors (hysterosalpingography guided)	Yes (151)	Yes	No	134/151 (88.2) normal	143/187 (76.4)	114/143 (79.2)

Bellingham, 1996 [42]	NR	17 (16 followed up) (NR)	NR	NR	Hysteroscopic division under US guidance	Yes	Yes	No	11/13 (84.6) normal	8/10 (80)	8/10 (80)

Roge et al., 1997 [9]	Retrospective	54 (52 followed up) (NR)	AFS	Mild (*n* = 12) Moderate (*n* = 12) Severe (*n* = 28)	Hysteroresectoscopy with resection electrode needle (under US guidance)	Yes	Yes	Yes	NR	34/52 (65.3)	24/34 (70.5)

Chen et al., 1997 [18]	NR	7 (31.14)	March	Severe	Hysteroresectoscopy with resection electrode needle	Yes	Yes	No	7/7 (100) normal	3/4 (75)	2/3 (66.6)

Feng et al., 1999 [13]	Retrospective cohort study	365 (33.8)	Sugimoto	NR	Hysteroscopy with microscissors and biopsy forceps	Yes	Yes	No	294/351 (83.7) normal	156/186 (83.8)	NR

Ozumba and Ezegwui, 2002 [32]	NR	50 (44 followed up)	NR	NR	Uterine sound and occasionally uterine dilators	Yes	Yes	No	34/44 (77.2) normal	4/44 (9)	NR

Orhue et al., 2003 [19]	NR	110 (26.7 ± 6.2)	NR	NR	Blind adhesiolysis under US guidance	Yes (51)	Yes	Yes (59)	32/51 (32.7) normal	14/51 (27.5)	6/14 (42.8)

Alborzi et al., 2003 [11]	Prospective	30 (30.4 up) (NR)	ASRM	Stage I (*n* = 11) Stage II (*n* = 13) Stage III (*n* = 6)	Hysteroscopy scissors (under vision of laparoscopy)	Yes	Yes	No	30/30 (100) normal	19/30 (63.3)	15/30 (50)

Zikopoulos et al., 2004 [16]	NR	46 (33.6)	AFS	Stage I (*n* = 6) Stage II (*n* = 25) Stage III (*n* = 15)	Resection using electrode needle (*n* = 21), bipolar electrosurgery system (*n* = 25)	Yes	Yes	No	13/14 (92.85) normal	35/46 (76.1)	20/46 (43.5)

Efetie, 2006 [12]	Retrospective	71 (27.97 ± 4.82)	NR	NR	Hysteroscopy, uterine sound	Yes	Yes	Yes	34/71 (47.9) normal	8/71 (11.3)	NR

Fumino et al., 2007 [44]	NR	47 (32.8)	AFS	I (*n* = 29) II (*n* = 14) III (*n* = 4)	Pushing via tip of hysteroscopy (*n* = 28) Ballooning at hysterosalpingography (*n* = 4), transcervical resectoscope, and mechanical D&C (*n*=13)	No	No	No	NR	20/47 (42.5)	NR

Shokeir et al., 2008 [41]	Retrospective	61 (31.5)	AFS	Stage II (*n* = NR) Stage III (*n* = NR)	Hysteroscopy with electrode needle	Yes (40)	Yes	No	NR	10/40 (40)	2/10 (20)

Yasmin et al., 2007 [33]	Descriptive study	20 (19 followed up) (26.1)	NR	Mild (*n* = 8) Moderate (*n* = 3) Severe (*n* = 9)	Blunt and resectoscopic dissection	Yes	Yes	Yes	18/19 (94.7) normal	2/19 (10.5)	1/2 (50)

Yu et al., 2008 [7]	Retrospective	85 (31.1)	ESH ESGE	Mild (*n* = 17) Moderate (*n* = 28) Severe (*n* = 40)	Hysteroscopy using electrode needle or loop	Yes	Yes	No	46/62 (74.2) improved	39/85 (45.88)	25/39 (64.1)

Pabuccu et al., 2008 [28]	Prospective, randomized trial	71 (group 1: 33.2 ± 1.5) (group 2: 32.6 ± 1.6)	AFS	Stage III	Sharp hysteroscopic division under US guidance	Yes	Yes	No	NR	Group 1: 17/36 (47.2) Group 2: 11/35 (31.4)	Group 1: 10/36 (27.7) Group 2: 7/35 (20)

Roy et al., 2010 [29]	Retrospective	96 (89 followed up) (28.4)	ESH, ESGE	I (*n* = 31) III (*n* = 40) IV (*n* = 18)	Hysteroscopic monopolar with Collin's knife	Yes	Yes	No	53/75 (70.67) improved	36/89 (44.4)	31/36 (86.1)

Salma et al., 2011 [34]	NR	60 (59 followed up) (29.3)	AFS	Severe	Hysteroscopy using scissors or electrode needle under direct vision	Yes	Yes	Yes	56/59 (94.9) normal	NR	NR

Myers and Hurst, 2012 [35]	Retrospective	12 (34.41)	AFS	Severe	Hysteroscopy scissors	Yes	Yes	Yes	12/12 (100) normal	6/8 (75)	4/6 (66.6)

Fernandez et al., 2012 [45]	Retrospective	23 (22 followed up) (34 ± 5.8)	ESHRE AFS	IV, severe	Hysteroscopy and bipolar electrosurgery system	No	No	No	1/24 (4.3%) (after 2 surgical procedures)	9/22 (40.9)	6/22 (27.2)

Mohamed et al., 2012 [39]	Retrospective	363 (130 followed up) (30.7 ± 5.6)	ESGE	Grade I (*n* = 79) Grade II (*n* = 167) Grade III (*n* = 103) Grade IV (*n* = 14)	Hysteroscopy with unipolar and bipolar electrosurgery	Yes	Yes	Yes	3/4 (75%) normal	40 (31.5%)	36/40 (90)

Yamamoto and Takeuchi, 2013 [36]	Retrospective	27 (35.4 ± 5.0)	AFS	Mild (*n* = 4) Moderate (*n* = 19) Severe (*n* = 4)	Hysteroscopic loop monopolar knife, Hegar's dilators (under US guidance)	Yes	Yes	No	27/27 (100) improved	14/27 (52.9 )	3/27 (11)

Lin et al., 2013 [27]	Retrospective cohort study	107 (30.4 ± 4.4)	AFS	Mild (*n* = 2) Moderate (*n* = 21) Severe (*n* = 5)	Hysteroscopic scissors	Yes (28)	Yes	Yes	18/28 (64.2) 8/28 (28.5) improved	NR	NR

Şendağ et al., 2013 [38]	NR	24 (30.5)	ESH	Grade 1 (*n* = 5) Grade 2 (*n* = 6) Grade 3 (*n* = 10) Grade 4 (*n* = 3)	Hysteroscopy with sharp scissors	Yes (11)	Yes	Yes	24/24 (100) normal	4/14 (28.5)	3/4 (75)

HT = hormonal therapy; IUD = intrauterine device; FC = Foley catheter; NR = not reported. “Yes” = studies that used IUD; “No” = studies that didn't used IUD.

**Table 2:** The classification systems of included studies.

American Fertility Society (AFS), 1988	Stage I, stage II, stage III
European Society of Hysteroscopy (ESH), 1989	Stage I, stage II, IIa, or III, stage IIIa, IIIb, or IV
European Society of Gynecological Endoscopy (ESGE), 1995	Stage I, stage II, IIa, or III stage IV, Va, or Vb
March, 1978	Mild, moderate, severe

**Table 3:** Summary of previously published studies that used various techniques of IUD therapy and ancillary treatment in patients with intrauterine adhesions.

Source, year	Type of IUD	Duration of IUD used	Hormone therapy	Foley catheter	Hyaluronic acid	Amnion graft
Caspi and Perpinial, 1975 [30]	Lippes loop	3 cycles	Yes	No	No	No
March and Israel, 1976 [31]	Lippes loop	2 months	Yes	Yes	No	No
March and Israel, 1981 [37]	Lippes loop	2 months	Yes	Yes	No	No
Ismajovich et al., 1985 [43]	NR	3 months	No	No	No	No
Fedele et al., 1986 [40]	NR	3 months	Yes	No	No	No
Valle and Sciarra, 1988 [8]	NR	3 months	Yes	No	No	No
Bellingham, 1996 [42]	Copper	3 months	Yes	No	No	No
Roge et al., 1997 [9]	NR	3 months	Yes	Yes	No	No
Chen et al., 1997 [18]	Multiload Cu 375	2 weeks	Yes	No	No	No
Feng et al., 1999 [13]	Multiload Cu 375	3 months	Yes	No	No	No
Ozumba and Ezegwui, 2002 [32]	Lippes loop	3 cycles	Yes	No	No	No
Orhue et al., 2003 [19]	Lippes loop	3 cycles	Yes	Yes	No	No
Alborzi et al., 2003 [11]	NR	1 month	Yes	No	No	No
Zikopoulos et al., 2004 [16]	Multiload Cu 375	1 month	Yes	No	No	No
Efetie, 2006 [12]	Lippes loop	3 months	Yes	No	No	No
Shokeir et al., 2008 [41]	NR	3 months	Yes	No	No	No
Yasmin et al., 2007 [33]	Lippes loop	3 cycles	Yes	Yes	No	No
Yu et al., 2008 [7]	Cu T	3 months	Yes	No	No	No
Pabuccu et al., 2008 [28]	Lippes loop	2 months	Yes	No	No	No
Roy et al., 2010 [29]	Cu T	30 days	Yes	No	No	No
Salma et al., 2011 [34]	Uterine-shaped	1 month	Yes	Yes	Yes	No
Myers and Hurst, 2012 [35]	Copper	4–10 weeks	Yes	Yes	No	No
Mohamed et al., 2012 [39]	Copper T 380A	1–3 months	Yes	Yes	No	Yes
Yamamoto and Takeuchi, 2013 [36]	NR	2 cycles	Yes	No	No	No
Lin et al., 2013 [27]	Copper coil	2 months	Yes	Yes	Yes	No
Şendağ et al., 2013 [38]	Cu T	1–3 months	Yes	Yes	No	No

NR = not reported. IUD = intrauterine device. “Yes” = studies that used IUD. “No” = studies that did not use IUD.
